# Massage Regulates Brain Plasticity in Chronic Sciatic Nerve Compression Injury Rats: A Study Based on Resting‐State Functional Magnetic Resonance Imaging

**DOI:** 10.1002/brb3.71545

**Published:** 2026-06-10

**Authors:** Lanting Huang, Xuekun Zhou, Lechun Chen, Chenyu Wang, Hao Wang, Huanzhen Zhang, Changzheng Jiang, Shuijin Chen, Jingjing Jiang, Qiangwen Xie, Zhigang Lin

**Affiliations:** ^1^ Fujian University of Traditional Chinese Medicine, Fuzhou Fujian Province China; ^2^ Fujian University of Traditional Chinese Medicine Affiliated Rehabilitation Hospital, Fuzhou Fujian Province China

**Keywords:** ALFF, brain function, CCI, injury, massage, pain, peripheral nerves, plasticity, rats, rs‐fMRI

## Abstract

**Background:**

Neuropathic pain (NP) is associated with maladaptive functional reorganization of the brain, yet the central mechanisms through which massage therapy exerts its analgesic effects remain poorly understood. This study aimed to investigate the impact of acupoint massage on spontaneous neural activity in a rat model of chronic constriction injury (CCI) of the sciatic nerve using resting‐state functional magnetic resonance imaging (rs‐fMRI).

**Methods:**

Male Sprague–Dawley rats (*N* = 45) were randomly allocated into three groups: control, CCI model, and CCI with massage intervention. The massage group received daily acupoint pressing therapy from postoperative day 4 to day 17. Mechanical paw withdrawal threshold (PWT) and thermal paw withdrawal latency (PWL) were assessed at baseline and on days 4, 7, 10, and 17 post‐modeling. rs‐fMRI scans were acquired at three time points (pre‐modeling, day 7, and day 17) using a 9.4T small‐animal MRI system. Whole‐brain amplitude of low‐frequency fluctuations (ALFF) was analyzed to evaluate spontaneous neural activity.

**Results:**

CCI modeling induced significant alterations in ALFF across multiple brain regions involved in sensory, affective, and cognitive processing, including the amygdala, hippocampus, insular cortex, and somatosensory cortex. Massage intervention produced a significant group × time interaction effect in the left hippocampus (voxel *p* < 0.005, cluster *p* < 0.05 FWE corrected). Specifically, at 7 days post‐modeling, ALFF values in the massage group were significantly lower than those in the model group (*p* = 0.0357), indicating early attenuation of CCI‐induced hippocampal hyperactivity. Behaviorally, massage intervention significantly elevated PWT and PWL from day 7 onward (*p* < 0.001), with sustained improvement through day 17.

**Conclusions:**

Massage therapy alleviates NP through modulation of spontaneous neural activity across multiple brain regions, with dynamic regulation of hippocampal plasticity emerging as a critical central mechanism. The early normalization of hippocampal hyperactivity may serve as a potential neuroimaging biomarker for massage‐mediated analgesia and provides experimental evidence supporting the clinical application of massage for NP management.

## Graphical abstract

Resting‐state functional magnetic resonance imaging analysis of chronic constriction injury rats after massage therapy.

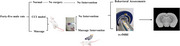

## 1 Introduction

Neuropathic pain (NP) is a pain condition directly caused by damage to or disease of the somatosensory system (Kocot‐Kępska et al. 2021). It is often manifested as spontaneous sensations such as burning, electric shock‐like, or numbness and is accompanied by sensory abnormalities (Manengu et al. 2025). The global prevalence of this condition in the general population is estimated to range from 7% to 10% (Hange et al. 2022). The symptoms are recurrent and protracted and significantly impair the patients’ quality of life (Colloca et al. 2017). The pathogenesis of NP encompasses multiple sensitization mechanisms spanning the peripheral and central nervous systems. Injury to peripheral nerves induces ectopic discharges (Chu et al. 2022), dysregulation of ion channels, and local release of pro‐inflammatory cytokines, which collectively evoke hyperalgesia (Chaban 2010; Finnerup et al. 2020) and facilitate the subsequent amplification of pain signals by injured neurons (He et al. 2017). Persistent abnormal afferent signals from the periphery trigger massive glutamate release from the spinal dorsal horn projection neurons (Jergova et al. 2025; Tian et al. 2024) and enhance the efficacy of synaptic transmission (Chen et al. 2022). This process is accompanied by glial cell activation, microglial release of pro‐inflammatory cytokines, and brain‐derived neurotrophic factor, which modulates the excitation–inhibition synaptic equilibrium and enhances plasticity, thereby driving central sensitization (Boakye et al. 2021). Complementarily, astrocytes regulate synaptic glutamate availability via the release of glutamate and ATP (Liu et al. 2021). The functional crosstalk between these glial subtypes engenders a positive feedback amplification circuit that consolidates the persistence of pain signaling and facilitates the transition to chronicity (Zhang et al. 2023; McKenzie et al. 2026). Beyond this glial orchestration, NP pathogenesis is further entrenched by a cascade of complementary mechanisms, including inflammatory signaling amplification (Ji et al. 2014), epigenetic reprogramming of pain‐related gene expression (Xiong et al. 2024), impairment of descending inhibitory pain pathways, and maladaptive structural or functional alterations in the sensory cortex subsequent to sustained pain exposure (Kim and Kim 2016). The treatment of NP is complex and challenging and remains a significant obstacle. Current primary therapies include pharmacological and non‐pharmacological approaches (He et al. 2024). Existing treatment strategies predominantly target peripheral mechanisms, but there are no established methods to effectively address the abnormal central brain activity sustained during chronic pain.

Tuina massage is a traditional manual therapy in Chinese medicine for alleviating chronic pain (Ma et al. 2023; Wu et al. 2019). The analgesic and anti‐inflammatory actions of massage are mediated through poly‐targeted, hierarchical mechanisms. Peripherally, massage abrogates Toll‐like receptor 4‐mediated signaling cascades to curb pro‐inflammatory cytokine release (Hu et al. 2024) while orchestrating microRNA networks to epigenetically restrain neuroinflammation and restore motor function and nociceptive thresholds in NP models (Wu et al. 2024). Spinally, massage suppresses microglial and astrocytic reactivity to dampen central sensitization and calibrates neurotransmitter dynamics and local inflammation via somatosensory afferent modulation of the central nervous system (Liu et al. 2022). Meta‐analytical evidence further confirms the effectiveness of massage across a spectrum of pain conditions – encompassing postoperative (Şengül and Çelik 2025), cancer‐related (Boyd et al. 2016), and chronic non‐specific low back pain (Yang et al. 2020). While the mechanistic basis of Tuina‐facilitated peripheral nerve repair has been extensively delineated, the extent to which it influences pain‐evoked maladaptive functional reorganization of the brain warrants further investigation.

Resting‐state functional magnetic resonance imaging (rs‐fMRI) is a powerful tool for assessing the brain's overall functional state (Chen et al. 2024). Low‐frequency amplitude reflects the intensity of local spontaneous neural activity; regional homogeneity estimates the functional consistency across brain regions; and graph‐theoretic analysis characterizes the whole‐brain functional connectivity and network topology. Together, these metrics provide a multidimensional perspective of brain functional remodeling (Zhang et al. 2024). Previous studies have demonstrated that under NP conditions, functional alterations occur not only in the primary somatosensory cortex but also in the higher order regions such as the anterior cingulate cortex (ACC), prefrontal cortex, and limbic system (Gao et al. 2025; Li et al. 2024). This suggests that pain disrupts networking in the whole brain. However, it is not clear whether massage techniques can alleviate abnormal brain activity and network imbalance caused by nerve injury and promote adaptive reorganization of brain function.

Therefore, this study established a chronic constriction injury (CCI) rat model to observe changes in pain thresholds before and after massage intervention post‐surgery while simultaneously detecting alterations in their brain's amplitude of low‐frequency fluctuations (ALFF). The study aimed to elucidate the effects of pain on the brain's central mechanisms and local brain activity from longitudinal and cross‐sectional perspectives. Furthermore, the study investigated whether massage intervention promotes recovery of neural functional activity in the brains of CCI rats, thereby providing neuroimaging evidence to support the clinical application of massage therapy.

## 2 Materials and Methods

### 2.1 Animals

Forty‐five male Sprague–Dawley rats, specific pathogen‐free grade, 8 weeks old, and weighing 180–220 g, were provided by the Experimental Animal Center of Fujian University of Chinese Medicine (License No. SYXK (Min)2019‐0007). All animals were housed at the Fujian University of Chinese Medicine Laboratory Animal Center under consistent environmental conditions, maintained on a 12‐hour light/dark cycle, with 3–5 rats per cage and free access to food and water. The rats were randomly divided into the following three groups: the control group (*n* = 15), the model group (*n* = 15) and the massage group (*n* = 15). Each group underwent one week of adaptation training. The experimental protocol was approved by the Animal Ethics Committee of Fujian University of Traditional Chinese Medicine (License No. FJTCMIACUC2024071).

### 2.2 CCI Model

The CCI model of NP was utilized in this study (Bennett and Xie 1988). Pain behavior tests were conducted 24 h prior to surgery to exclude rats with abnormal baseline values, followed by a 12‐hour fast. Rats in the massage group and model group were anesthetized with an intraperitoneal injection of 4% sodium pentobarbital (0.2 mL/100 g). The right hindlimb muscles were bluntly dissected to expose the sciatic nerve. Four catgut sutures were ligated with moderate tension to induce slight limb twitching and were spaced 1.0–1.5 mm apart. The surgical site was irrigated with saline postoperatively, followed by layered closure and local application of penicillin to prevent infection. Successful modeling was confirmed when rats exhibited pronounced plantar flexion and varus deformity of the right hindfoot and protective behaviors such as licking and non‐weight‐bearing of the affected limb, especially 1‐day post‐modeling (Bennett and Xie 1988).

### 2.3 Massage Intervention

**FIGURE 1:**
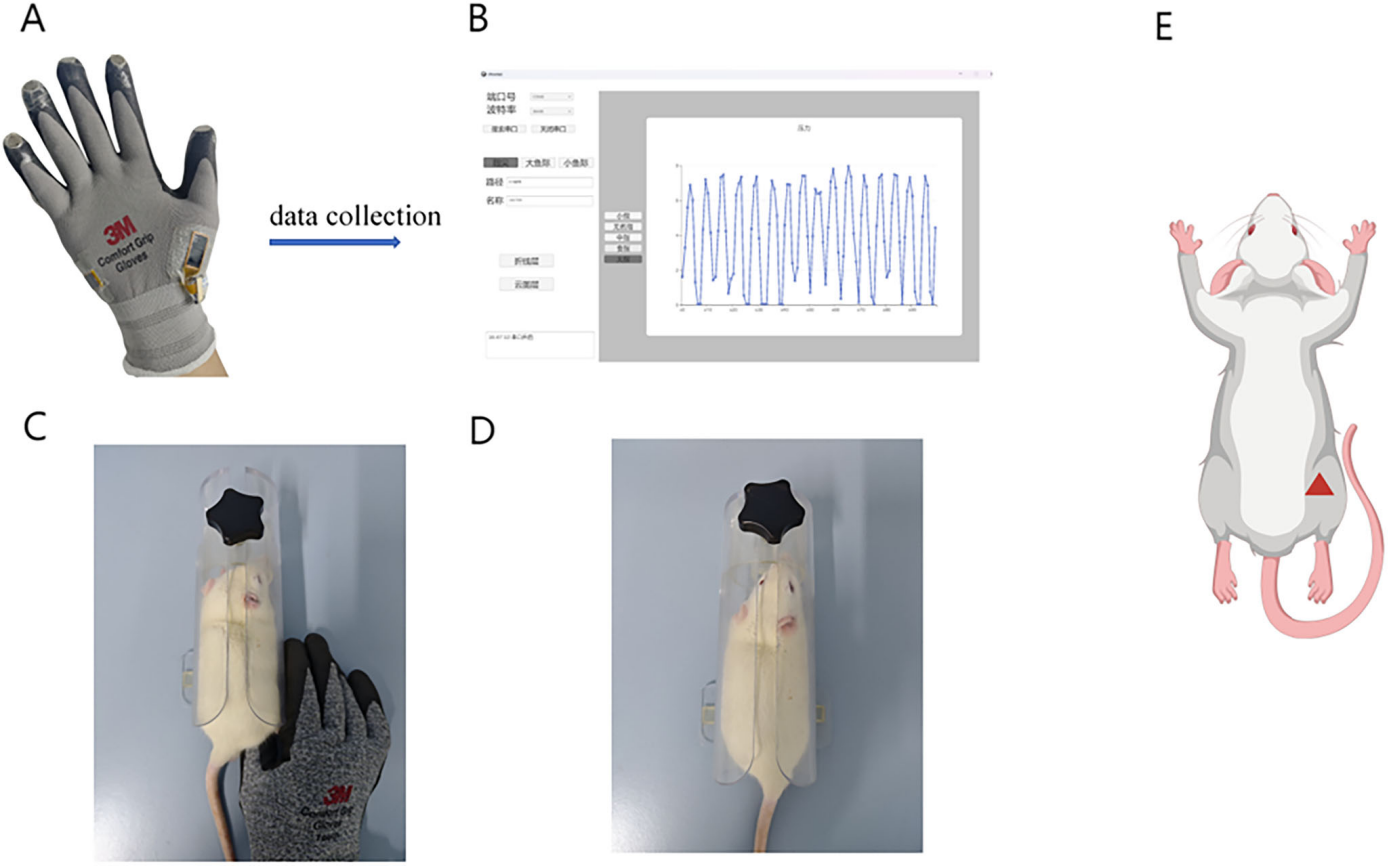
(A) Intelligent massage glove. (B) Data collection interface. (C) Massage intervention. (D) No intervention. (E) Massage intervention location.

On the fourth postoperative day, rats in the massage group underwent massage and acupressure intervention. Prior to intervention, rats were placed for 15 min in a massage restraint device for acclimatization. The manual technique was performed using our team's proprietary multi‐signal fusion massage technique quantification system (Patent No.: ZL202123343241.0). This system comprises an intelligent massage glove, a multi‐sensor array, a data acquisition system, and a real‐time display interface. The glove's fingers feature single‐point force sensors arranged in a three‐dimensional surround configuration to precisely capture the intensity and frequency of finger‐pressing techniques. Sensor data were processed and transmitted to a computer‐based visualization interface. Operators adjust the pressing technique to ensure precision by monitoring real‐time data, graphical trends, and analysis results. After wearing smart gloves, operators apply pressure with their thumb tips firmly against the rat's Shu‐side Weizhong acupoint, gradually increasing force from light to heavy in a steady and sustained manner. Simultaneously, they observe parameters on the glove interface to make mechanical adjustments. Massage intervention was administered once daily at 5N force, 120 times/min, and 10 min per session (Huang et al. 2024). Rats in the model group were similarly placed in the massage fixture daily without manual intervention, while the control group did not receive any intervention (Figure 1).

### 2.4 Behavioral Tests

Paw withdrawal threshold (PWT): Changes in the mechanical paw‐withdrawal threshold were observed in the three groups of rats at baseline before modeling and on days 4, 7, 10, and 17 after modeling. The rats were placed in a transparent plastic box (20 × 25 × 15 cm) for 30 min of adaptation. The box top featured five 5mm diameter ventilation holes and rested on a custom‐made wire rack. Then, sequential stimuli were applied to the plantar surface of the right hind paw using von Frey filaments, and the force value from stimulus onset to the appearance of paw‐lifting or paw‐withdrawal responses was recorded (Chaplan et al. 1994). Each paw was assessed five times. Then, the highest and lowest values were discarded, and the mean value of the middle three readings was recorded as the result. The tests were conducted at least 5 min apart on the same rat.

**FIGURE 2:**
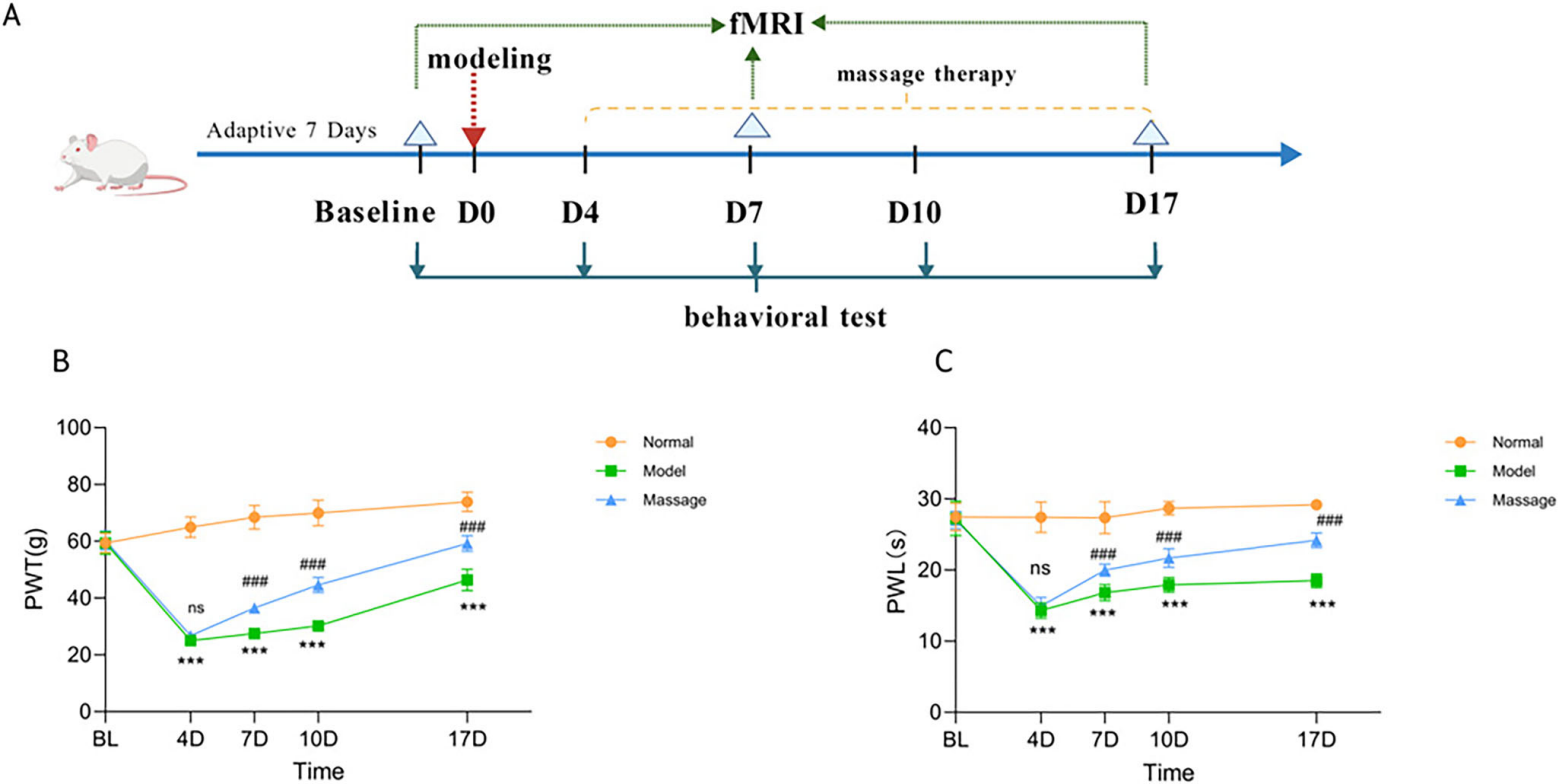
(A) Flowchart of experimental strategy. (B) Comparison of PWL values between the three rat groups at different time points. (C) Comparison of PWT values between the three rat groups at different time points. PWL: paw withdrawal latency; PWT: paw withdrawal threshold; BL: baseline; behavioral testing was conducted before modeling and on days 4, 7, 10, and 17 post‐modeling. Compared with the control, ***p < 0.001; compared with the model, ^###^ *p* < 0.001.

Paw withdrawal latency (PWL): Changes in paw withdrawal latency were observed in the three groups of rats at baseline before modeling and on days 4, 7, 10, and 17 after modeling. Rats were acclimatized for 30 min in a transparent plastic box (20 × 25 × 15 cm) with five 5‐mm diameter ventilation holes on the top that was placed on a custom‐made glass platform. Thermal stimulation was applied to the plantar surface of the right hind paw using a thermal pain tester. The latency was recorded from stimulus onset to paw withdrawal (Dirig et al. 1997). Each paw was evaluated five times. The highest and lowest values were discarded, and the mean of the middle three readings was recorded for each rat. The rats were given at least a 5‐minute interval between tests (Figure 2A).

### 2.5 fMRI Data Acquisition

The fMRI scanning and imaging were performed using a 9.4T small‐animal MRI system (Bruker, Germany) at the following three time points: pre‐modeling, 7 days post‐modeling, and 17 days post‐modeling. Rats were anesthetized with 3% isoflurane prior to scanning (Stanton et al. 2021). They were secured in a prone position on the MRI scanner bed using a dental bar and bilateral ear bars. A dedicated surface coil was fixed to the rat's head. A water‐circulation heating system was used to maintain constant body temperature. Anesthesia with 1.5% isoflurane was maintained throughout scanning and real‐time monitoring of body temperature, respiration, and heart rate (Kannurpatti et al. 2008). The REST (T2star_FID_EPI, Exp3) acquisition parameters were as follows: TR: 2.13444 s; TE: 20 ms; FA: 90°; FOV: 32 × 32 mm; and Matrix: 128 × 128; Slice parameters were as follows: Slice thickness: 0.5 mm; Number of slices: 41; Inter‐slice spacing: 0.10003 mm; Image quality and acceleration parameters: Voxel size: 0.25 × 0.25 × 0.6 mm (Vrooman et al. 2025). The T2 (T2_TurboRARE, Exp2) acquisition parameters were as follows: TR: 4.68289 s; TE: 37.69 ms; FA: 90°; FOV: 32 × 32 mm; and Matrix: 256 × 256; Slice parameters were as follows: Slice thickness: 0.5 mm; Number of slices: 41; Inter‐slice spacing: 0.10003 mm; Image quality and acceleration parameters were as follows: Voxel size: 0.125 × 0.125 × 0.6 mm.

### 2.6 fMRI Data Processing

Data preprocessing was performed using the Statistical Parametric Mapping toolbox (SPM12; http://www.fil.ion.ucl.ac.uk/spm/) based on MATLAB R2022b. First, raw DICOM images were converted to NIfTI format using dcm2niix with original voxel dimensions scaled by a factor of 10. The first 10 time points were discarded to ensure magnetization equilibrium. The remaining functional volumes underwent slice timing correction (interleaved order, starting with even slices) and realignment to the mean functional image to correct for head motion. Spatial normalization was executed using the ANTs toolkit (https://github.com/ANTsX/ANTs) via a two‐step registration procedure: the mean functional image was linearly co‐registered to the individual T2‐weighted structural image, which was subsequently normalized to the symmetric Wistar rat brain template (Johnson et al. 2021) using nonlinear registration. These transformations were applied to the functional images, which were then resampled to a voxel size of 1.5 × 1.5 × 1.5 mm^3^. Post‐normalization processing differed slightly between metrics: for ALFF analysis, images were spatially smoothed with a 3‐mm full‐width at half‐maximum Gaussian kernel. Finally, all data underwent linear detrending and regression of nuisance covariates, including white matter signals, cerebrospinal fluid signals, and Friston 24 motion parameters, followed by temporal band‐pass filtering. All 45 rats completed the three scanning sessions without any exclusions due to motion or artifacts, yielding 15 subjects per group at each time point for final analysis.

### 2.7 Statistical Analysis

This study used mixed‐effects analysis of variance to evaluate differences in all fMRI metrics across different groups and time points. For voxel‐based analyses, statistical results underwent dual threshold correction, wherein an uncorrected *p*‐value was first set at the voxel level, followed by FWE correction at the cluster level (*p* < 0.05). For significant clusters, the mean value of each cluster was extracted for each subject. If the mixed ANOVA revealed significant main effects or interaction effects, post hoc tests were conducted to identify the specific sources of differences. Behavioral data were analyzed using the repeated measures ANOVA, whereas one‐way ANOVA was used for between‐group comparisons. A *p*‐value <0.05 was considered statistically significant.

## 3 Results

### 3.1 Behavioral Assessment Findings

The pre‐treatment PWT and PWL values were not significantly different between the three groups. However, from modeling to day 4, the PWT and PWL values were significantly lower in the model group and massage group compared with the control group. On day 4 post‐modeling, the PWT and PWL values for the model group were significantly lower compared to the control group (*p* < 0.001) but were not significantly different than the massage group. However, on days 7, 10, and 17 post‐modeling, PWT and PWL values in the massage group were significantly higher than in the model group (*p* < 0.001) (Figure 2B,C).

### 3.2 Group Main Effects of ALFF

**FIGURE 3:**
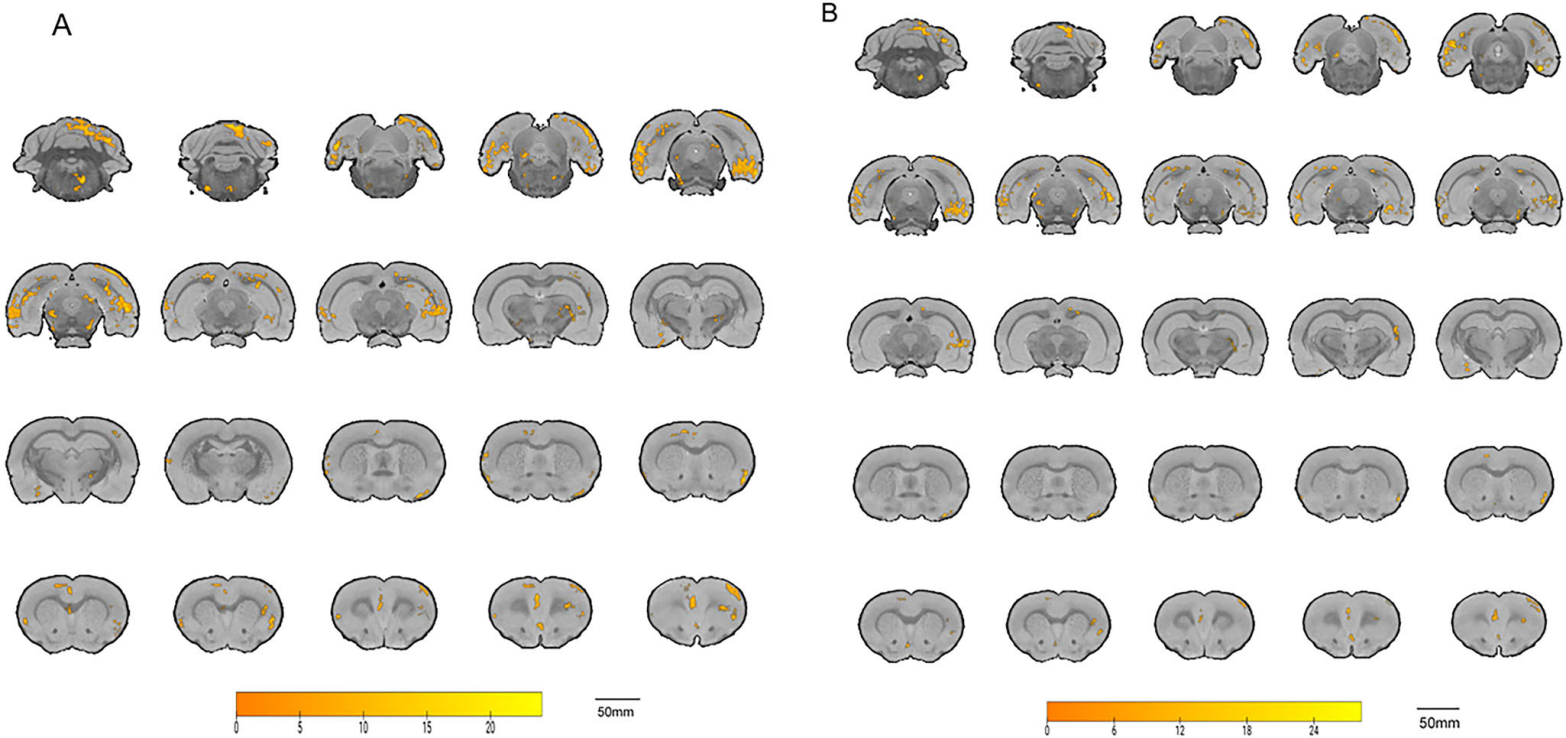
(A) Visualization of significant differences in the group main effects. Higher brightness indicates greater significance. The illustration shows a 10× magnification. The scale bar represents 50 mm. (B) Visualization of significant differences in time main effect clusters. Higher brightness indicates greater significance. The illustration shows a 10× magnification. The scale bar represents 50 mm.

The group main effects of ALFF were significantly different across all three groups, independent of time points in 40 brain regions based on the following threshold parameters: voxel *p* < 0.001; cluster *p* < 0.05 FWE corrected (Table 1 and Figure 3A).

**TABLE 1:** Group main effect ALFF values based on a mixed ANOVA for clusters with significant differences.

Cluster No.	Brain regions	MNI coordinates (mm)			*F* value	Cluster size
		X	Y	Z		
1	AA__Amygdalar_Area_left	−4.42	−1.815	−3.71	10.0623	150
2	PIR__Piriform_Area_right	4.43	−2.565	0.19	16.0483	627
3	moV__Motor_Root_of_the_Trigeminal_Nerve_left	−2.62	−2.565	−9.71	9.9958	167
4	HB__Hindbrain_Uncharted_right	1.28	−1.665	−10.31	14.5878	186
5	HY__Hypothalamus_Uncharted_left	−1.57	−2.415	−4.01	12.5563	72
6	ll__Lateral_Lemniscus_right	2.78	−1.215	−8.66	13.4452	87
7	cbp__Cerebellar_Peduncles_left	−2.17	−1.215	−7.01	12.2033	246
8	ENT__Entorhinal_Area_left	−5.62	2.535	−7.91	24.0886	3619
9	ENT__Entorhinal_Area_right	5.93	−0.915	−7.16	22.2152	2480
10	PIR__Piriform_Area_right	5.78	−0.615	1.24	18.4677	408
11	SNr__Substantia_Nigra_Reticular_Part_right	2.18	−0.915	−6.71	14.1656	144
12	PIR__Piriform_Area_left	−6.37	−0.315	0.34	13.6153	130
13	ZI__Zona_Incerta_Uncharted_left	−2.62	−0.315	−4.01	9.2466	80
14	ZI__Zona_Incerta_Uncharted_right	2.33	−0.165	−3.26	13.1941	143
15	ECT__Ectorhinal_Area_right	6.83	1.335	−5.51	14.6482	473
16	AI__Agranular_Insular_Area_left	−5.47	1.485	2.74	11.7686	201
17	Isocortex__Isocortex_Uncharted_left	−0.07	0.435	3.04	13.8138	75
18	ZI__Zona_Incerta_Uncharted_right	3.98	0.585	−4.46	14.718	104
19	CUN__Cuneiform_Nucleus_left	−1.87	1.335	−8.21	13.1959	202
20	fi__Fimbria_right	5.78	2.985	−4.01	16.2039	498
21	PrCnF__Precuneiform_Nucleus_left	−2.47	0.735	−6.71	12.7919	169
22	TH__Thalamus_Uncharted_right	2.78	1.485	−5.66	11.1615	71
23	AI__Agranular_Insular_Area_right	4.58	1.335	3.34	13.9744	89
24	VISC__Visceral_Area_left	−7.42	1.635	−1.61	12.3217	172
25	VIS__Visual_Areas_right	5.48	5.085	−7.46	24.3288	3416
26	cc__Corpus_Callosum_right	4.43	1.935	2.14	11.504	100
27	SCs__Superior_Colliculus_right	2.33	2.535	−6.71	12.7131	194
28	ILA__Infralimbic_Area_left	−0.37	3.435	3.49	12.9052	380
29	cc__Corpus_Callosum_right	3.23	2.685	3.04	11.7613	123
30	SCs__Superior_Colliculus_left	−2.62	2.685	−6.11	9.8338	101
31	HPF__Hippocampal_Formation_Uncharted_right	4.28	3.885	−6.71	15.1238	607
32	MO__Somatomotor_Areas_right	4.28	4.485	3.49	11.0337	295
33	SS__Somatosensory_Areas_right	5.33	5.385	−3.41	15.5202	181
34	HPF__Hippocampal_Formation_Uncharted_right	3.68	4.185	−5.81	14.2411	83
35	ACA__Anterior_Cingulate_Area_left	−0.37	4.635	1.84	11.4444	114
36	MO__Somatomotor_Areas_left	−0.97	5.235	3.04	10.2623	72
37	VIS__Visual_Areas_left	−5.32	5.085	−7.76	9.488	70
38	MO__Somatomotor_Areas_left	−1.57	5.385	1.24	12.1186	273
39	cc__Corpus_Callosum_left	−2.02	5.385	−5.81	11.5088	81
40	PTLp__Posterior_Parietal_Association_Areas_right	4.43	5.985	−4.16	12.8031	74

*Note*: voxel: *p* < 0.001; cluster *p* < 0.05 FWE‐corrected.

### 3.3 Time Main Effects of ALFF

The time main effects of ALFF were significantly different across all three groups at the three indicated time points in 40 brain clusters based on the following thresholds: voxel *p* < 0.001; cluster *p* < 0.05 FWE‐corrected (Table 2 and Figure 3B).

**TABLE 2:** Time main effect ALFF values based on a mixed ANOVA for clusters with significant differences.

Cluster No.	Brain regions	MNI coordinates (mm)			*F* value	Cluster size
		X	Y	Z		
1	AA__Amygdalar_Area_left	−3.82	−2.415	−3.41	11.3624	55
2	SOC__Superior_Olivary_Complex_left	−2.47	−2.565	−9.71	10.1585	65
3	PIR__Piriform_Area_right	4.43	−2.565	0.19	20.3682	89
4	HB__Hindbrain_Uncharted_right	1.28	−1.665	−10.31	16.2777	36
5	ENT__Entorhinal_Area_left	−5.92	2.385	−8.66	28.8114	1451
6	AA__Amygdalar_Area_left	−4.12	−1.815	−3.56	13.1193	33
7	cbp__Cerebellar_Peduncles_left	−2.17	−1.215	−7.01	11.8699	40
8	SNr__Substantia_Nigra_Reticular_Part_right	2.18	−0.915	−6.71	15.9606	81
9	ENT__Entorhinal_Area_right	5.93	−0.915	−7.16	25.2876	1034
10	HPF__Hippocampal_Formation_Uncharted_right	4.58	−0.165	−6.41	16.3993	115
11	PIR__Piriform_Area_right	6.23	−0.465	0.79	17.5279	125
12	OLF__Olfactory_Areas_Uncharted_left	−0.37	−0.765	1.99	15.2144	35
13	PIR__Piriform_Area_left	−6.37	−0.315	0.34	17.6308	37
14	Isocortex__Isocortex_Uncharted_left	−0.07	0.435	3.19	15.3435	48
15	ZI__Zona_Incerta_Uncharted_right	3.98	0.585	−4.46	16.0662	45
16	HPF__Hippocampal_Formation_Uncharted_right	6.38	0.885	−5.51	15.2988	154
17	CUN__Cuneiform_Nucleus_left	−1.87	1.335	−8.21	16.5575	45
18	MRN__Midbrain_Reticular_Nucleus_Uncharted_left	−2.02	0.585	−6.71	13.2394	64
19	AI__Agranular_Insular_Area_right	5.33	0.885	2.14	12.6819	47
20	cc__Corpus_Callosum_right	5.93	1.785	−4.01	13.7773	34
21	PAR__Parasubiculum_right	5.33	1.935	−7.91	15.7061	90
22	Brain__Brain_Uncharted_left	−3.37	1.635	−6.71	13.9467	35
23	PAR__Parasubiculum_left	−4.27	2.385	−7.76	16.9862	75
24	SS__Somatosensory_Areas_right	4.73	2.385	2.14	12.7773	40
25	PAR__Parasubiculum_right	4.13	3.285	−8.36	13.9023	39
26	ILA__Infralimbic_Area_left	−0.37	2.835	3.34	13.1627	162
27	cc__Corpus_Callosum_right	3.23	2.685	3.04	12.1879	32
28	VIS__Visual_Areas_right	5.48	5.085	−7.46	23.8379	1877
29	cc__Corpus_Callosum_right	6.08	2.535	−4.01	15.6001	64
30	SCs__Superior_Colliculus_left	−2.62	2.835	−6.26	10.2337	42
31	HPF__Hippocampal_Formation_Uncharted_right	4.28	3.885	−6.71	14.0648	89
32	SS__Somatosensory_Areas_right	4.73	5.085	2.74	13.5364	151
33	TEa__Temporal_Association_Areas_left	−4.72	4.185	−7.76	15.1713	46
34	cc__Corpus_Callosum_left	−3.52	4.785	−7.46	16.5705	78
35	cc__Corpus_Callosum_right	2.93	4.935	−6.41	12.3989	47
36	cc__Corpus_Callosum_left	−3.97	5.085	−6.56	14.4734	66
37	cc__Corpus_Callosum_right	2.63	5.085	−4.61	14.3216	34
38	RSP__Retrosplenial_Area_left	−2.32	5.535	−6.41	12.9578	42
39	MO__Somatomotor_Areas_left	−1.57	5.385	1.24	12.8482	51
40	RSP__Retrosplenial_Area_right	1.58	5.685	−5.21	14.3877	33

*Note*: voxel: *p* < 0.001; cluster: *p* < 0.05 FWE‐corrected.

### 3.4 Interaction Effects

**FIGURE 4:**
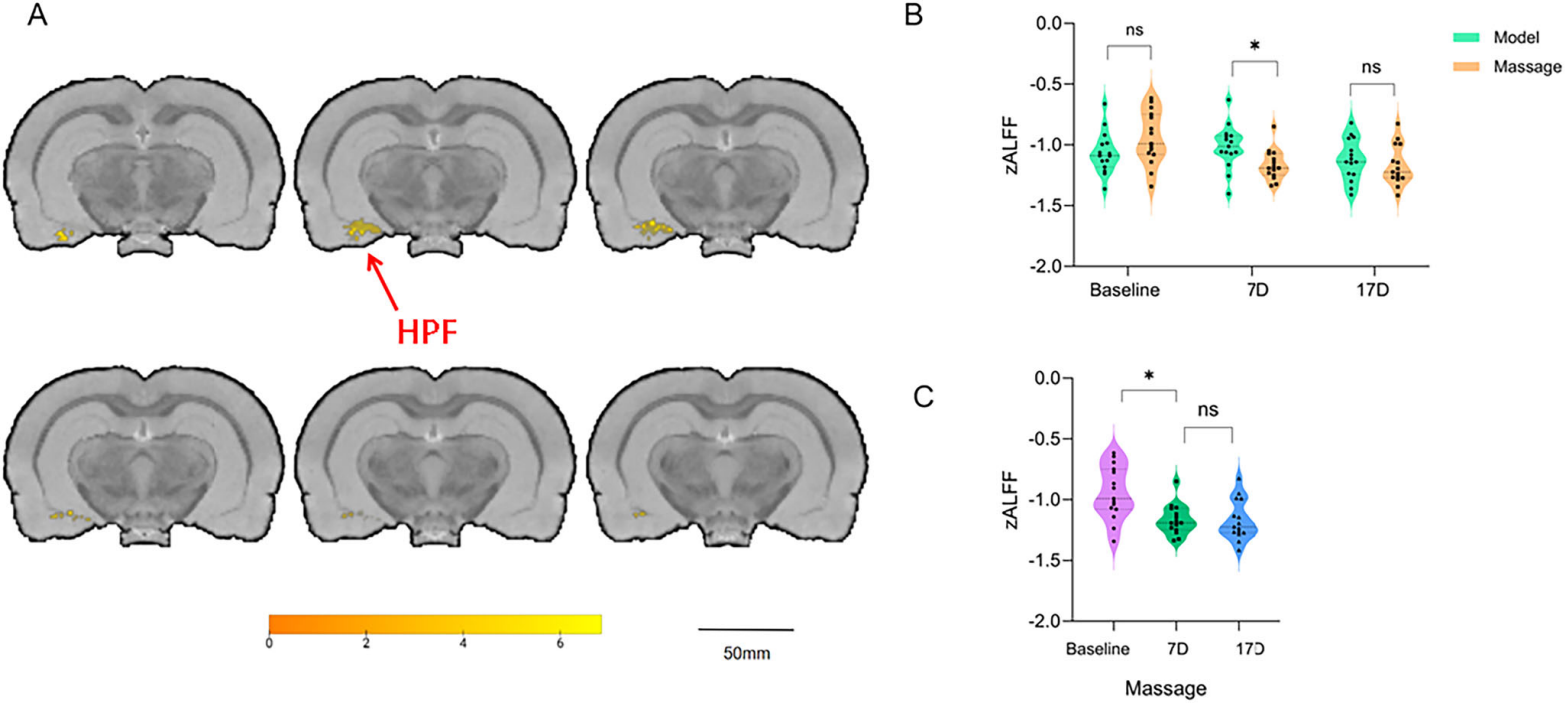
(A) Visualization of clusters with significant interaction effects. Higher brightness indicates greater significance. Red arrows point to the left hippocampal region. HPF: hippocampal region. The illustration shows a 10× magnification. The scale bar represents 50 mm. (B) The zALFF values in the left hippocampus for the three groups of rats at three time points, compared with the model, *p < 0.05 (baseline, day 7, and day 17). (C) The zALFF values in the left hippocampus of the massage group at three time points, compared with baseline, *p < 0.05 (baseline, day 7, and day 17).

Next, we analyzed the interaction effects between group and time point. As shown in Figure 4, we observed significant differences in the interaction effects of the left hippocampal region. Then, we set the following thresholds: voxel: *p* < 0.005; cluster: *p* < 0.05 FWE‐corrected. Based on the interaction effect findings, we investigated whether massage intervention affected the longitudinal and lateral changes in spontaneous neural activity in the hippocampal region of the postoperative rats. The statistical results indicated no significant difference between the model group and the massage group at baseline. At 7 days post‐modeling, the ALFF value in the massage group was significantly lower than the ALFF value in the model group (*p* = 0.0357), thereby indicating a significant early effect of the massage intervention. At 17 days post‐modeling, although the ALFF values in the model group remained numerically higher than those in the massage group, the difference was no longer statistically significant. The massage group showed a significant decrease in ALFF values compared to baseline at 7 days post‐modeling (*p* = 0.0231) and remained stable from day 7 to day 17 post‐modeling (Table 3 and Figure 4A–C).

**TABLE 3:** Clusters with significant differences in interaction effects ALFF values based on mixed ANOVA.

Cluster No.	Brain regions	MNI coordinates (mm)			*F* value	Cluster size
		X	Y	Z		
1	HPF__Hippocampal_Formation_Uncharted_left	−4.42	−1.365	−4.76	6.9207	97

*Note*: voxel: *p* < 0.005; cluster: *p* < 0.05 FWE‐corrected.

## 4 Discussion

Tuina massage is a traditional Chinese therapy using non‐pharmacological, non‐invasive manual techniques to stimulate and regulate central sensitization. Nociceptive signaling in NP propagates via central pain pathways to elicit aberrant functional alterations across multiple brain regions, while massage exerts its modulatory influence by targeting the function of these pain‐associated cerebral networks. The central analgesic mechanisms of Massage are characterized by hierarchical modulation of the pain matrix, the default mode network (DMN), and descending pain modulatory circuitry (Chmiel and Kurpas 2026). Massage attenuates hyperactivation in core nociceptive regions – namely, the ACC, insula, thalamus, and primary sensorimotor cortex (Li et al. 2008; Song et al. 2024) – while partially normalizing aberrant intra‐ and inter‐network functional connectivity involving the DMN and sensorimotor systems in chronic pain states (Zhang et al. 2015). Additionally, manual stimulation potentiates periaqueductal gray ‐thalamic functional coupling to recruit descending inhibitory pathways and engages limbic substrates – including the nucleus accumbens and amygdala – to confer emotional‐reward modulation (Lyu et al. 2025). Cumulatively, these observations demonstrate that massage orchestrates a comprehensive recalibration of sensory, cognitive, and affective pain domains by reshaping integrative information dynamics within brain networks.

The ALFF derived from rs‐fMRI constitutes a principal metric of spontaneous synchronous neuronal firing intensity, synaptic plasticity, and neurovascular coupling within circumscribed brain regions (Lv et al. 2020; Rocchi et al. 2022). Signal amplitude oscillations in ALFF directly reflect concurrent states of neuronal excitability and functional brain activity. In pain research, rs‐fMRI leverages the blood oxygen level‐dependent effect – whereby changes in regional cerebral perfusion indirectly signal central neural activity (Gay et al. 2014) – and ALFF has thus been widely applied to characterize central nervous system dysfunction in chronic pain conditions. Studies have shown the following: Chronic pain and depression comorbidity in mice alters neuronal activity across multiple brain regions, with elevated ALFF values in the hippocampus, occipital cortex, substantia nigra, dentate gyrus, entorhinal cortex, temporal cortex, and auditory cortex, and reduced ALFF values in the insular cortex and amygdala; electroacupuncture treatment decreases ALFF values in multiple amygdala‐related regions, and the most pronounced modulatory effects are observed in the basolateral amygdala (Yin et al. 2025). Wu and colleagues found that chronic compression of the dorsal root ganglion (CCD) surgery on the right hindlimb of rats significantly reduced ALFF values in the left amygdala–hypothalamic region, the left amygdala's piriform cortex, the left descending cortical pathway, the globus pallidus, and the left dentate gyrus. This indicates that neural injury reduced spontaneous neuronal activity in the contralateral hemisphere. Subsequently, Tuina manipulation significantly increased ALFF values in these brain regions compared to the CCD group, thereby suggesting restoration of neuronal activity (Wu et al. 2023). Xing and colleagues reported that ALFF values were reduced in the contralateral somatosensory cortex following peripheral nerve injury, thereby indicating diminished spontaneous (excitatory) neuronal activity. However, ALFF values increased post‐intervention because of enhanced neuronal activity as a result of peripheral nerve repair and regeneration. This suggests that manual intervention can influence pain perception through functional integration of the neuronal system, thereby exerting analgesic effects (Xing et al. 2021). Therefore, by adopting ALFF as an observational index, this study aimed to objectively characterize the regulatory influence of massage on the central mechanisms subserving NP.

This study investigated the dynamic effects of a CCI model of the sciatic nerve and massage intervention on spontaneous neural activity in the rat brain. Analysis of group main effects showed overall differences in ALFF across multiple brain regions among the three groups, including the left and right medial entorhinal cortex, left amygdala, left parahippocampal gyrus, right piriform cortex, right visual cortex, somatosensory cortex, left motor cortex, left cerebellar peduncle, right substantia nigra reticularis, and left cingulate gyrus. These regions are extensively involved in emotion processing, memory encoding, sensory integration, and motor coordination. Therefore, different pain states may significantly influence related brain functional activities. The main effect of time demonstrated that ALFF dynamically changed over time in multiple brain regions, including the medial olfactory cortex, hippocampal regions, amygdala, piriform lobe, substantia nigra reticularis, visual cortex, somatosensory cortex, corpus callosum, and cerebellar peduncles. This suggests that the CCI surgery and postoperative recovery process induce significant plastic changes in the brain. Further analysis showed that 19 core brain regions exhibited significant effects in the group and time main effects, which could be parcellated into three interconnected functional networks. The limbic‐emotional network comprised the left amygdalar area, right hippocampal formation, bilateral piriform areas, right agranular insular cortex, left infralimbic area, and an uncharted region of the left isocortex. The pain‐sensory integration network included bilateral entorhinal areas, the right somatosensory cortex, right visual areas, the left superior colliculus, and the right corpus callosum. The pain‐signaling and motor‐coordination network consisted of the right hindbrain, left cerebellar peduncles, right substantia nigra pars reticulata, right zona incerta, left cuneiform nucleus, and left somatomotor areas. These regions form an interconnected functional network, thereby indicating that NP induces dynamic and widespread neural activity in the brain.

Our data revealed significant ALFF alterations across three functionally distinct yet interconnected networks, collectively underscoring that chronic pain may extend far beyond a mere sensory disturbance to encompass pronounced affective, mnemonic, and motor dysregulation (Fiúza‐Fernandes et al. 2025). Within the limbic‐emotional network, hyperactivation of the left amygdala amplifies pain‐related distress and hyperalgesia, while concurrent changes in the right hippocampus and piriform cortices disrupt memory consolidation and cognitive function (McCarberg et al. 2019); furthermore, the right agranular insula integrates interoceptive signals with emotional awareness to directly fuel anxiety (Labrenz et al. 2022), and left infralimbic dysfunction impairs fear extinction and autonomic regulation (Ma et al. 2024), with alterations in the uncharted isocortex further indicating that chronic pain impacts limbic cortical fields beyond conventional anatomical boundaries (Naylor et al. 2019). In parallel, the pain‐sensory integration network exhibited coordinated changes indicative of disrupted nociceptive processing and aberrant multimodal integration. Specifically, the entorhinal cortices were implicated in encoding the contextual and spatial features of pain rather than its raw emotional valence (Gim et al. 2024), while somatosensory changes reflected deficits in sensory‐discriminative processing and maladaptive reorganization (Moseley and Flor 2012). Furthermore, synchronized visual and superior colliculus alterations signify enhanced visuo‐somatosensory cross‐modal integration that likely sustains hyperarousal and vigilance (Kragel et al. 2021), all compounded by disrupted interhemispheric sensory‐integrative communication via the corpus callosum. Complementing these findings, the pain‐signaling and motor‐coordination network shows ALFF changes consistent with a pathological shift from inhibitory to facilitatory descending control that potentiates ascending nociceptive transmission, a process further reinforced by compromised diffuse noxious inhibitory control in the hindbrain (Lockwood and Dickenson 2020; Patel and Dickenson 2020) and aberrant activity in the primarily inhibitory zona incerta, which directly facilitates pain and motor avoidance (Moon and Park 2017), while alterations in the cerebellar peduncles disrupt spinocerebellar proprioceptive input and sensorimotor coordination (Li et al. 2024), and changes in the somatomotor areas provide the cortical substrate for pain‐induced motor dysfunction (George et al. 2025). Moreover, specific subcortical modulatory pathways, including the substantia nigra pars reticulata (Han et al. 2024) and a nigro‐subthalamo‐parabrachial circuit (Jia et al. 2022), further mediate the integration of nociception and motor output. These findings indicate substantial spatial overlap between the main effects of group and time, suggesting that pain status and disease progression jointly drive widespread neural adaptation. However, main effects only capture global mean differences and thus fail to delineate the differential regulatory patterns exerted by massage intervention across distinct temporal stages of the disease course. Consequently, the group × time interaction effect constitutes the critical analytical focus for elucidating the central‐specific modulatory mechanisms of massage intervention in NP.

Interaction effects exhibited significant differences in the left hippocampal region. This indicated that unlike the broad group or time main effects observed in other brain areas, the pattern of functional activity changes in the hippocampus specifically depended on the combined effects of different groups at different time points. The specificity of these interaction effects further suggests a mechanism by which massage intervention remodels brain function in NP. To investigate the effects of massage intervention on neural activity at different time points, the interaction effects in the left hippocampal region were further analyzed. Our results showed no significant differences between the model group and the massage group at baseline. However, at 7 days post‐modeling, CCI‐induced pain caused abnormal ALFF values in the model group, but the massage group exhibited significantly reduced ALFF values compared to the model group. This suggests that pain induces excessive neural activity in the left hippocampal region, whereas massage effectively reverses pain‐induced brain dysfunction at an early stage. By day 17 post‐modeling, the massage intervention maintained stable neuromodulatory effects. The model group remained more active than the massage group but gradually converged toward the massage group through self‐recovery and regulation, with no significant difference in ALFF values between the two groups. ALFF values in the massage group decreased significantly compared to baseline, 7 days post‐modeling. No significant differences were observed between ALFF values from days 7 to 17 post‐modeling. This indicated that massage intervention demonstrated excellent early efficacy under pathological pain conditions and maintained stable long‐term therapeutic effects. Behavioral findings aligned with this trend. At day 4 post‐modeling, CCI rats exhibited significantly lower mechanical PWTs and thermal paw‐withdrawal latencies than the control group, thereby confirming successful CCI modeling. In contrast, the massage group exhibited a sustained recovery in PWT and PWL from days 7 to 17 post‐modeling and approached control levels by day 17. This indicated that massage effectively ameliorates pain behavior, and its trajectory was highly consistent with the dynamic changes in the hippocampal ALFF values. These findings demonstrate the long‐term effects of the massage intervention in normalizing the brain dysfunction induced by NP.

The hippocampus is the core structure of the limbic system and is indispensable for learning, memory, and affective processing (Garman et al. 2023). Chronic pain impairs hippocampal‐related memory formation, especially suppressing spatial memory formation (Xia et al. 2020). Extensive evidence indicates that chronic pain is not merely a static sensory phenomenon but rather a learning and memory disorder accompanied by alterations in central plasticity (Saffarpour et al. 2017). Pain states can be conceptualized as intense aversive affective internalization processes, where the hippocampus participates in encoding and consolidating pain‐related contextual, emotional, and sensory information, subsequently leading to hyperalgesia, anxiety‐like behaviors, and memory impairment (Sumizono et al. 2022). A previous study showed that the hippocampal region of healthy volunteers was activated in response to painful stimuli (Ezzati et al. 2014). Left hippocampal volume positively correlates with mechanical pain sensitivity, whereas greater hippocampal damage correlates with reduced sensitivity to mechanical pain (Ayoub et al. 2024). Under painful conditions, reduced hippocampal neurogenesis, synaptic plasticity, and neuronal communication manifest as peripheral behavioral deficits, thereby indicating the crucial role of the hippocampus in pain experience (Tyrtyshnaia et al. 2025). In this study, the hippocampus consistently exhibited significant effects in group and time analyses, thereby confirming its critical role in NP, a finding consistent with our group's prior mechanistic work. Specifically, we have established that the massage intervention activates hippocampal SIRT1/BDNF/TrkB signaling (Wang et al. 2025), leading to an upregulation of synaptic markers NR2B and PSD95 (Huang et al. 2024). This pathway drives the structural and functional restoration of neurons within the hippocampal CA1 and CA3 regions, as evidenced by enhanced dendritic spine density, increased synapse number, and the reversal of synaptic ultrastructural pathology. These morphological improvements correspond to the alleviation of nociceptive behaviors and the attenuation of secondary depressive‐like symptoms. Accordingly, we propose that these neuroplastic adaptations serve as the mechanistic foundation for massage‐induced relief of hyperalgesia subsequent to chronic neuropathic injury. The mechanical stimulation of massage potentially alleviates NP through its rhythmic and benign sensory input by regulating the autonomic nervous system balance and promoting hippocampal neuroplasticity. Therefore, it mitigates pain‐related emotions and inhibits the formation of pain memories. This represents one of the key central mechanisms underlying the analgesic effects of the massage intervention.

## 5 Conclusion

In the context of NP, the limbic‐emotional, sensory‐integrative, and transmission‐motor networks constitute a functionally coupled “sensory‐emotional‐motor” circuit that supports multidimensional pain coding. Massage exerts analgesia via multi‐target modulation of this circuit, restoring nodal homeostasis, reversing maladaptive plasticity, and driving the adaptive reorganization of central pain pathways. The left hippocampus serves as a critical hub, encoding aversive pain memory while integrating sensory, affective, and motor dimensions. Convergent imaging and behavioral data suggest that massage dynamically regulates hippocampal memory reconsolidation and emotional adaptation, dampening pain memory reinforcement, correcting maladaptive behaviors, and reestablishing central homeostatic plasticity. These findings identify left hippocampal hub‐mediated circuit remodeling as the core central mechanism of massage analgesia, offering key experimental evidence for its clinical use in NP.

Several limitations should be acknowledged. First, the pain behavioral assessment was incomplete, covering only mechanical and thermal pain without evaluating spontaneous or cold pain. Second, although massage parameters were standardized, the optimal pressure, duration, frequency, and acupoint selection remain undefined, limiting the generalizability of the findings. Third, the resting‐state fMRI experimental design requires additional control groups, such as normal rats receiving massage and sham‐operated rats. Fourth, although neuroimaging showed that massage modulates hippocampal plasticity and resting‐state activity and previous experiments linked massage to the hippocampus, changes in hippocampal molecular signaling pathways were not examined for bidirectional validation. Finally, only male rats were used, precluding analysis of sex differences. Future studies should include more comprehensive pain behavioral assessments, refined control groups, investigation of region‐specific brain pathways, and optimization and validation of massage parameters to more precisely elucidate the central mechanisms of massage analgesia.

## Author Contributions

**Xuekun Zhou**, **Chenyu Wang**, and **Hao Wang** performed experiments; **Lanting Huang** and **Changzheng Jiang** analyzed MRI data; **Lanting Huang** drafted the manuscript; Experimental supervision: **Zhigang Lin**, **Qiangwen Xie**; Statistical analysis supervision: **Huanzhen Zhang**, **Jingjing Jiang**; Manuscript revision: **Lechun Chen**, **Shuijin Chen**. All authors read and approved the final manuscript.

## Funding

This study was supported by Provincial Natural Science Foundation Project 2023J06037: Investigation of the Mechanism by Which Traditional Chinese Medicine Tuina Modulates Synaptic Plasticity in the VPL‐S1 Neural Circuit of Lumbar Disc Herniation. Principal Investigator: Zhigang Lin. National Natural Science Foundation of China 82575244: Mechanism of Pressing‐Kneading Manipulation in Modulating Munc18‐1/SNARE Complex to Intervene Presynaptic Homeostasis in the Dorsal Horn of Spinal Cord in Rats with Lumbar Disc Herniation. Principal Investigator: Zhigang Lin. Provincial Health Commission Mid‐Career Talent Development Project: Mechanism of Tuina in Alleviating Neuropathic Pain by Modulating Peripheral Sensitization in CCI Rats via P2×3‐CaMKII‐CREB Pathway. Principal Investigator: Lechun Chen. National Natural Science Foundation of China 82575250 Mechanism of Massage Point‐Pressure Technique in Inhibiting Peripheral Sensitization in Rats with Neuropathic Pain via Piezo2 Channel‐Mediated Myelin Regeneration of Aβ Fibers Principal Investigator: Lechun Chen.

## Conflicts of Interest

The authors declare no conflicts of interest.

## Data Availability

All the data information is in the manuscript.
